# Supplementary material for: I-Shaped Dimers of a Plant Chloroplast FOF1-ATP Synthase in Response to Changes in Ionic Strength
Source: Int J Mol Sci. 2023 Jun 27;24(13):10720. doi: 10.3390/ijms241310720 (PMC10341776; doi:10.3390/ijms241310720)
Supplement: Supplementary file 1 [file ijms-24-10720-s001.zip › ijms-2412952-supplementary.pdf]

Supplementary Information

I-shaped dimers of a plant chloroplast F_OF_1-ATP synthase in response to changes in ionic strength

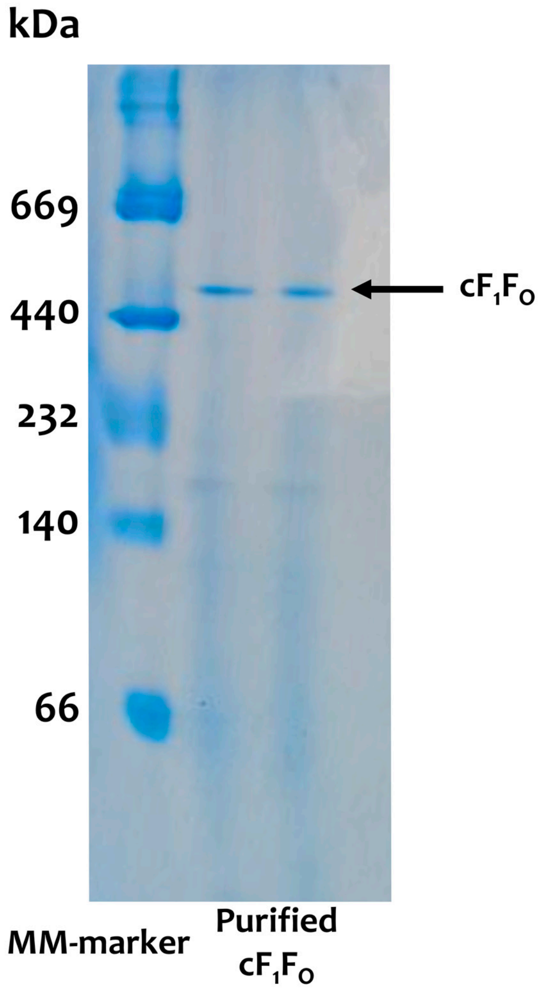

Figure S1. Characterization of the purified cF_OF_1 by BN-PAGE (6 – 18 %). MM-marker: 66, 140, 232, 440, 669 kDa. Purified samples of cF_OF_1 showed the band between 440 and 669 kDa. The molecular weight of the cF_OF_1 complex is ~595 kDa. Marker proteins are water soluble but cF_OF_1 is a partly membrane protein complex and it also has a detergent belt, therefore different migration distance could be observed. A single band demonstrates that cF_OF_1 is intact.

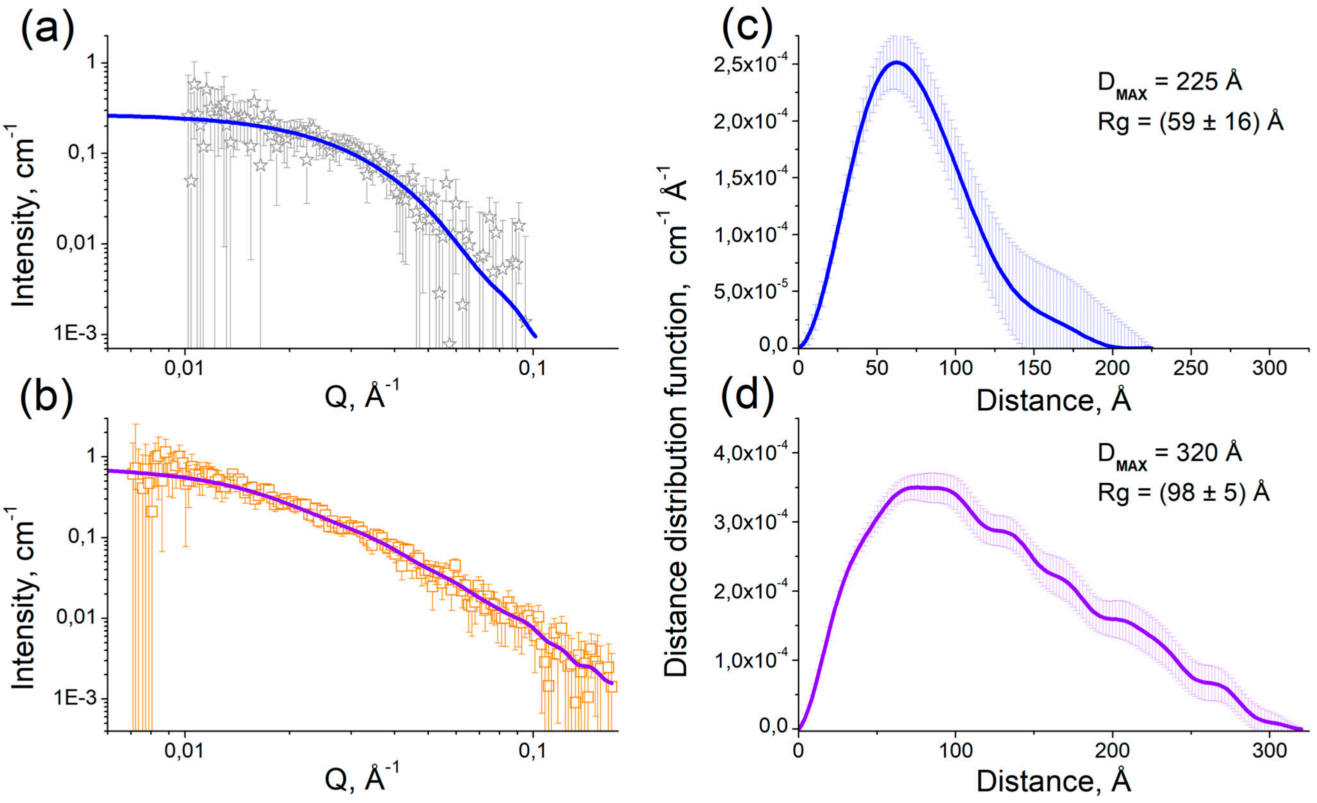

Figure S2. SANS characterization of cF_OF_1: (**a**) SANS experimental data for AEX-purified cF_OF_1 in H_2O-buffer (hollow grey stars) and a regularized fit (blue line); (**b**) SANS experimental data for cF_OF_1 at 300 mM NaCl in 93% D_2O-buffer (hollow orange squares) and a regularized fit (purple line); (**c**) Pair-distance distribution function P(r) for cF_OF_1 in H_2O-buffer; (**d**) Pair-distance distribution function P(r) for cF_OF_1 at 300 mM NaCl in 93% D_2O-buffer.

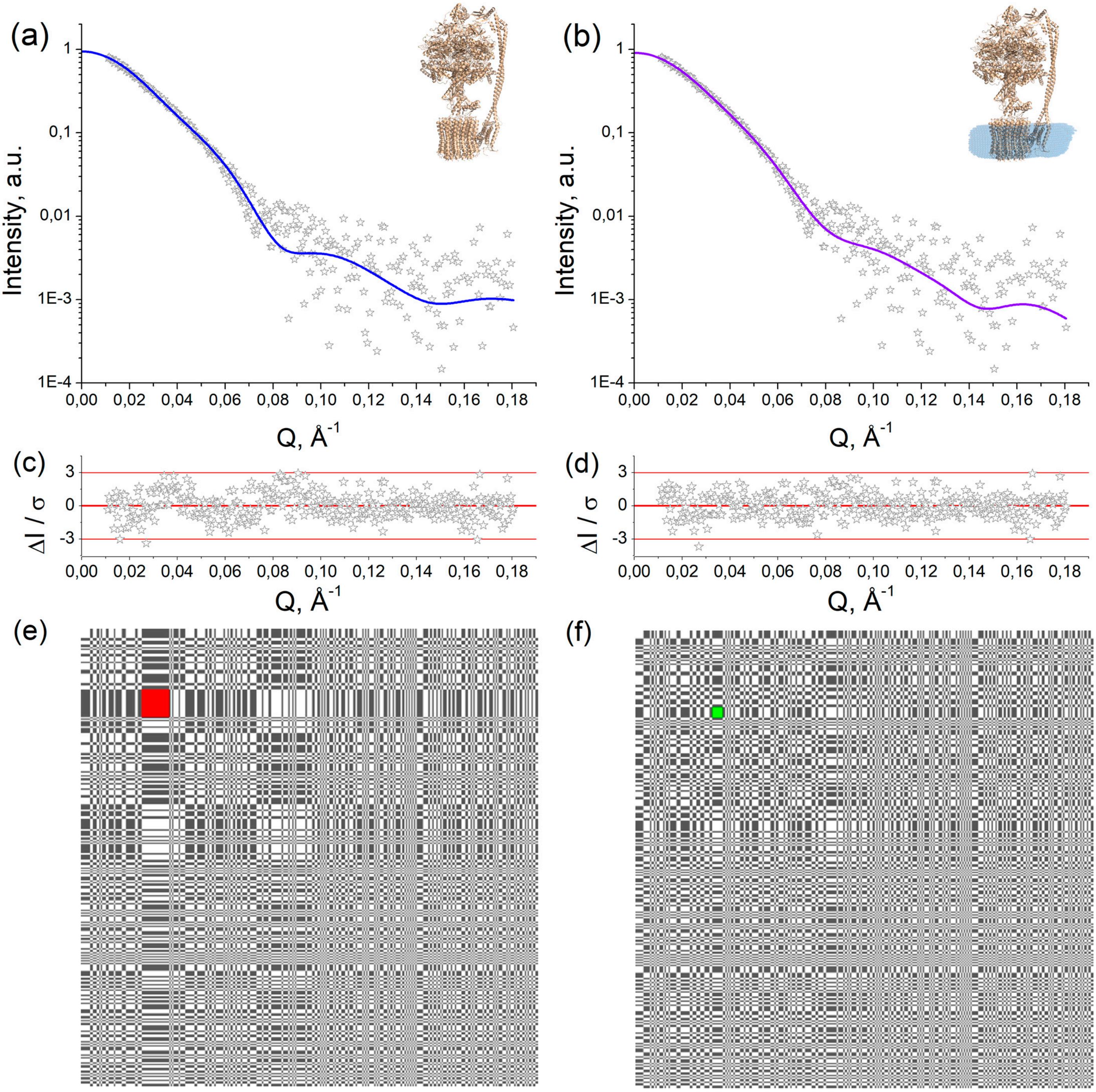

Figure S3. Influence of a detergent belt on quality of SAXS data approximation: (**a**) SAXS experimental data for cF_OF_1 (hollow grey stars) and an approximation (blue line, χ^2 = 1.35) obtained by CRYSOL for the model of cF_OF_1 without a detergent belt (PDB ID: 6FKF); (**b**) SAXS experimental data for cF_OF_1 the same as in panel (**a**) (hollow grey stars) and an approximation (blue line, χ^2 = 1.15) obtained by CRYSOL for model of protein with detergent belt built by MEMPROT; (**c**) Relative residues of the fit shown in panel (**a**); (**d**) Relative residues of the fit shown in panel (**b**); (**e**) Correlation map (P-value 0.000156) for the fit shown in panel (**a**); (**f**) Correlation map (P-value 0.745484) for the fit shown in panel (**b**).

(a)

# model	HDOCK		PDBePISA				$D_{m\text{-}m}$, Å	SAXS data approximation for cF_OF_1				
	Docking score	Confidence score	interface area, Å²	ΔG, kcal/mol	N_{HB}	N_{SB}		150 mM NaCl	250 mM NaCl	300 mM NaCl	350 mM NaCl	450 mM NaCl
**1**	-154.82	0.5241	1910,5	-0,3	13	7	168	χ^2 = 1.41<br>α_2 = 65 ± 2 %	χ^2 = 1.13<br>α_2 = 18 ± 2 %	χ^2 = 1.22<br>α_2 = 39 ± 2 %	χ^2 = 1.07<br>α_2 = 30 ± 2 %	χ^2 = 1.34<br>α_2 = 60 ± 2 %
**2**	-152.11	0.5105	1046,9	1,7	6	6	159	χ^2 = 1.41<br>α_2 = 62 ± 2 %	χ^2 = 1.13<br>α_2 = 17 ± 2 %	χ^2 = 1.23<br>α_2 = 37 ± 2 %	χ^2 = 1.07<br>α_2 = 28 ± 2 %	χ^2 = 1.34<br>α_2 = 57 ± 2 %
**3**	-141.80	0.4591	1888,2	0,6	13	5	166	χ^2 = 1.42<br>α_2 = 64 ± 2 %	χ^2 = 1.13<br>α_2 = 18 ± 2 %	χ^2 = 1.22<br>α_2 = 39 ± 2 %	χ^2 = 1.08<br>α_2 = 29 ± 2 %	χ^2 = 1.35<br>α_2 = 59 ± 2 %
**4**	-139.19	0.4462	1776,5	-4,3	10	6	203	χ^2 = 1.29<br>α_2 = 73 ± 2 %	χ^2 = 1.11<br>α_2 = 21 ± 2 %	χ^2 = 1.25<br>α_2 = 43 ± 2 %	χ^2 = 1.00<br>α_2 = 35 ± 2 %	χ^2 = 1.20<br>α_2 = 68 ± 2 %
**5**	-139.18	0.4461	1218,3	4,8	17	4	206	χ^2 = 1.26<br>α_2 = 77 ± 2 %	χ^2 = 1.10<br>α_2 = 23 ± 2 %	χ^2 = 1.28<br>α_2 = 44 ± 3 %	χ^2 = 0.96<br>α_2 = 38 ± 2 %	χ^2 = 1.17<br>α_2 = 71 ± 2 %
**6**	-138.84	0.4444	1225,8	4,2	13	3	207	χ^2 = 1.26<br>α_2 = 77 ± 2 %	χ^2 = 1.10<br>α_2 = 23 ± 2 %	χ^2 = 1.28<br>α_2 = 44 ± 3 %	χ^2 = 0.95<br>α_2 = 38 ± 2 %	χ^2 = 1.17<br>α_2 = 71 ± 2 %
**7**	-137.62	0.4384	1347,6	2	9	8	206	χ^2 = 1.25<br>α_2 = 76 ± 2 %	χ^2 = 1.10<br>α_2 = 23 ± 2 %	χ^2 = 1.27<br>α_2 = 44 ± 3 %	χ^2 = 0.96<br>α_2 = 37 ± 2 %	χ^2 = 1.16<br>α_2 = 71 ± 2 %
**8**	-131.93	0.4106	1033,1	-4,1	8	0	194	χ^2 = 1.25<br>α_2 = 75 ± 2 %	χ^2 = 1.09<br>α_2 = 23 ± 2 %	χ^2 = 1.27<br>α_2 = 44 ± 3 %	χ^2 = 0.96<br>α_2 = 37 ± 2 %	χ^2 = 1.16<br>α_2 = 70 ± 2 %
**9**	-129.08	0.3969	1219,3	-0,8	10	6	209	χ^2 = 1.28<br>α_2 = 75 ± 2 %	χ^2 = 1.10<br>α_2 = 22 ± 2 %	χ^2 = 1.27<br>α_2 = 44 ± 3 %	χ^2 = 0.98<br>α_2 = 36 ± 2 %	χ^2 = 1.19<br>α_2 = 70 ± 2 %
**10**	-127.96	0.3916	1033,9	2,3	9	5	151	χ^2 = 1.36<br>α_2 = 62 ± 2 %	χ^2 = 1.12<br>α_2 = 17 ± 2 %	χ^2 = 1.24<br>α_2 = 36 ± 2 %	χ^2 = 1.05<br>α_2 = 28 ± 2 %	χ^2 = 1.29<br>α_2 = 57 ± 2 %

(b)

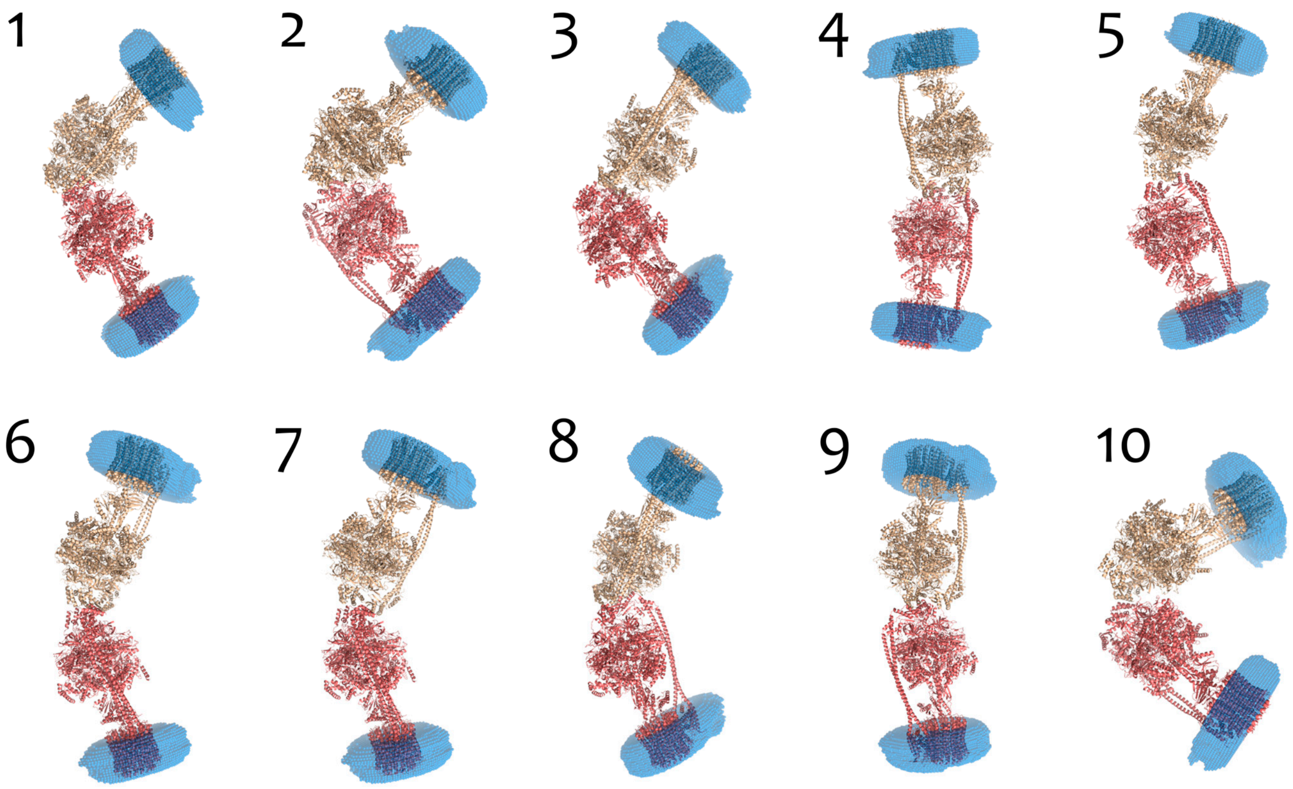

Figure S4. Models of F_1/F_1-interface dimers of cF_OF_1 based on the top 10 HDOCK predictions: (**a**) Table of scoring parameters and SAXS data treatment parameters obtained for the top 10 HDOCK predictions (see section *Macromolecular docking* in Materials and Methods). Table columns are: internal HDOCK docking quality estimations (Docking score and Confidence score), parameters of macromolecular interfaces estimated using the PDBePISA web-server (interface area, Gibbs energy ΔG, numbers of hydrogen bonds N(HB) and salt bridges N(SB)), distances $D_{m\text{-}m}$ between centers of mass of monomers in the dimers of cF_OF_1, and parameters of approximations of SAXS data obtained at 150, 250, 300, 350, and 450 mM NaCl (χ^2 values and volume fractions of dimers α_2 obtained by the fit in program OLIGOMER); (**b**) Representations of cF_OF_1 dimers based on the top 10 HDOCK predictions. The models contain detergent belts obtained by the program MEMPROT.

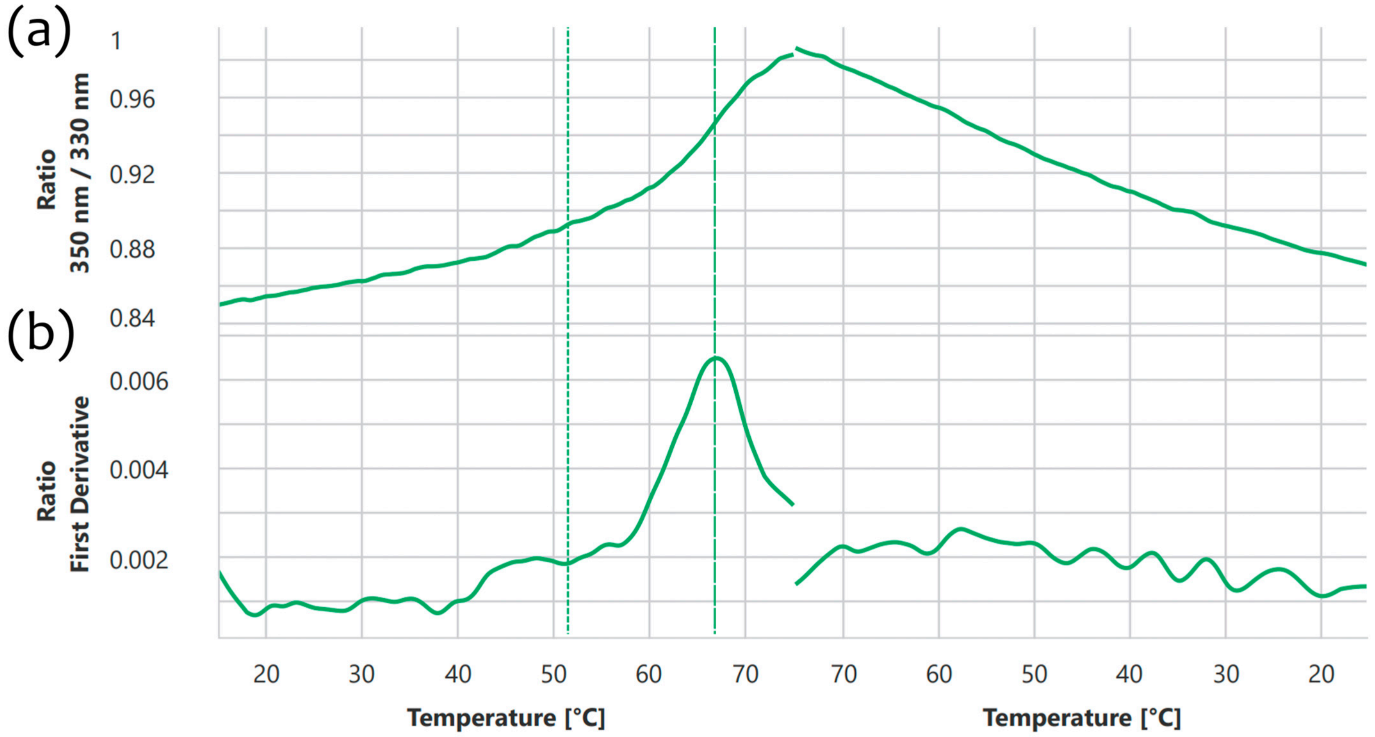

Figure S5. Thermal unfolding and refolding graphs measured by nanoDSF method. Vertical short dash line corresponds to T_{onset}, dash line shows melting temperature T_m: (**a**) Ratio of fluorescence intensity at 330 nm and 350 nm for temperature range from 15 °C to 75 °C; (**b**) First derivative of the 330 nm/350 nm fluorescence intensity ratio.

Table S1. SAXS experimental details and data evaluation summary.

***(a)* Sample details**[1]

	ATPs AEX	*ATPs 150 mM NaCl*	*ATPs 250 mM NaCl*	*ATPs 300 mM NaCl*	*ATPs 350 mM NaCl*	*ATPs 450 mM NaCl*
Description of sequence	ATP synthase complex from *Spinacia Oleracea* (full structure: 6FKF), one complex contains: 3 α (UniProt ID P06450), 3 β (UniProt ID P00825), γ (UniProt ID P05435), delta (UniProt ID P11402), ε (UniProt ID P00833), a (UniProt ID P06451), b (UniProt ID P06453), b′ (UniProt ID P31853), 14 c (UniProt ID P69447).					
ε280 (M^{-1} cm^{-1})	α: 26820, β: 17880, γ: 10430, δ: 5960, ε: 11000, a: 42400, b: 9970, b′: 4470, c: 1490, full complex: 196 790					
Partial specific volume	0.7286 cm^3/g					
Complex volume	722670 Å^3					
Molecular mass	597.24 kDa					
Sample concentration	5.0 mg/ml					
Solvent composition	30 mM HEPES (pH 8.0), 2 mM $MgCl_2$, 0.04% *tpcc-α-M*,					
	~300 mM NaCl	150 mM NaCl,	250 mM NaCl,	300 mM NaCl,	350 mM NaCl,	450 mM NaCl,

(*b*) SAS data collection parameters

Instrument	Rigaku MicroMax-007 HF (MIPT, Dolgoprudny, Russia)
Wavelength	1.5406 Å
Beam geometry	Size (FWHM beam diameter at sample position): 0.3 mm; Sample-to-detector distance: 2.0 m
Sample configuration	Glass capillaries with diameters of ~1.5 mm
Q-measurement range	0.007 – 0.215 Å^{-1}
Q-scaling method	Calibration standard: silver behenate powder
Exposure time	5400 sec for each sample
Sample temperature	20 °C

(*c*) Software employed for SAS data reduction, analysis and interpretation

Data averaging and subtraction, Guinier analysis	PRIMUSqt from ATSAS
Calculation of ε from sequence	ProtParam: https://web.expasy.org/protparam/
Calculation of volume from chemical composition	Peptide Property Calculator: http://biotools.nubic.northwestern.edu/proteincalc.html
P(*r*) analysis	GNOM from ATSAS
Atomic structure modelling	HDOCK web-server; MEMPROT 2.2; CRYSOL 2.0 (command line mode)
Molecular graphics	PyMOL 1.9.x

(*d*) Structural parameters

		ATPs AEX	*ATPs 150 mM NaCl*	*ATPs 250 mM NaCl*	*ATPs 300 mM NaCl*	*ATPs 350 mM NaCl*	*ATPs 450 mM NaCl*
P(*r*) analysis	I(0) (a.u.)	1.03 ± 0.02	1.35 ± 0.04	1.00 ± 0.03	1.02 ± 0.03	1.39 ± 0.05	1.66 ± 0.06
	Rg (Å)	76.5 ± 2.2	104.7 ± 5.0	82.5 ± 3.7	90.3 ± 5.1	110.3 ± 7.8	129.9 ± 5.4
	D_{max} (Å)	285.0	402.16	333.5	382.0	453.0	465.0
	Q-range (Å^{-1})	0.0085 – 0.1355	0.0065 – 0.1355	0.0065 – 0.1355	0.0065 – 0.1355	0.0065 – 0.1355	0.0065 – 0.1355
	Q_{min} D_{max} / π	0.77	0.83	0.69	0,79	0.93	0.96
	Total quality estimate (GNOM)	0.6756	0.6556	0.6460	0.5497	0.6647	0.6468

[1] These values were calculated from the protein sequence without taking into account possible ligands (ADP, ATP, etc.) or detergent molecules.

Gunier analysis					
I(0) (a.u.)	1.05 ± 0.03	1.33 ± 0.03	0.97 ± 0.02	0.97 ± 0.03	1.30 ± 0.03
Rg (Å)	75.9 ± 3.0	96.0 ± 2.9	73.7 ± 1.7	78.3 ± 3.1	88.7 ± 2.8
Q Rg – range	0.64 – 1.29	0.72 – 1.30	0.48 – 1.29	0.51 – 1.29	0.58 – 1.29

(e) Atomistic modelling

	ATPs AEX	*ATPs 150 mM NaCl*	*ATPs 250 mM NaCl*	*ATPs 300 mM NaCl*	*ATPs 350 mM NaCl*
Q-range ($Å^{-1}$)	0.011 – 0.1805	0.0075 – 0.1805	0.0070 – 0.1805	0.0065 – 0.1805	0.0065 – 0.1805
**Method**	CRYSOL 2.0 (command line mode)				
Any measures of model precision	Protein model (PDB ID: 6FKF) without detergent belt				
Solvation shell Δϱ	0.020 e / $Å^3$				
Ra	1.640 Å				
Volume	757369 $Å^3$				
χ^2 value	1.347				
**Method**	MEMPROT; CRYSOL 2.0 (command line mode)				
Any measures of model precision	Protein model (PDB ID: 6FKF) with pseudo-atomic detergent belt obtained using MEMPROT. Para detergent belt: adaptive shape algorithm type 2 (MBJP), a = 32.4 Å, b = 7.0 Å, t = 6.5 Å, e = 1.0, #				
Solvation shell Δϱ	0.075 e / $Å^3$				
Ra	1.400 Å				
Volume	980265 $Å^3$				
χ^2 value	1.149				
**Method**	OLIGOMER				
Any measures of model precision	Experimental data were fitted by a set of curves calculated using CRYSOL 2.0 (command line mode) and dimers of cF_OF_1. Calculations of a set of form-factors were performed for the models of mono meric cF_OF_1 containing detergent belts obtained by MEMPROT using the optimal parameters of the obtained for the AEX-purified protein: Ra = 1.4 Å, Δϱ(shell) = 0.075 e / $Å^3$, monomer volume = 9				
Volume fraction of the cF_OF_1 dimers		0.733 ± 0.022	0.214 ± 0.022	0.431 ± 0.024	0.350 ± 0.020
χ^2 value		1.29	1.11	1.25	1.00

(f) Data and model deposition IDs *

	ATPs AEX	*ATPs 150 mM NaCl*	*ATPs 250 mM NaCl*	*ATPs 300 mM NaCl*	*ATPs 350 mM NaCl*
	SASDRR8	SASDRS8	SASDRT8	SASDRU8	SASDRV8

Text document S1. Estimation of the inter-monomer distance in ATP-synthase dimers.

For a mixture of various types of particles with different volumes (*V_j*), scattering length densities ($\Delta\bar{\rho}_j$) and gyration radii (Rg_j) scattering intensity dependence on the scattering vector absolute value using Guinier approximation (at "small" q, when $q \times Rg \ll 1$) is given by:

$$I(q) = \sum_j n_j V_j^2 \Delta\bar{\rho}_j^2 \left(1 - \frac{{Rg_j}^2}{3} q^2\right) \quad \text{(S1)}$$

where n_j is the concentration of the jth type of particles respectively. In case when there are two types of particles: monomers (j = 1) and dimers (j = 2), the following relations take place:

$$\begin{cases} V_2 = 2V_1, \\ m_2 = 2m_1 \\ \Delta\bar{\rho}_1 = \Delta\bar{\rho}_2, \\ Rg_2^2 = {Rg_1}^2 + (D_{m-m}/2)^2, \\ n_1 = c(1-\alpha)/m_1, \\ n_2 = c\alpha/m_2, \end{cases} \quad \text{(S2)}$$

where c is the total protein mass concentration, a is the dimer volume fraction, m_j is the certain type particle mass, $D_{m\text{-}m}$ is the inter-monomer distance between the mass centers within a dimer.

Using relations (2), equation (1) can be rearranged as:

$$I(q) = n_1 V_1^2 \Delta\bar{\rho}_1^2 (1+\alpha)\left(1 - \left({Rg_1}^2 + \frac{2\alpha}{1+\alpha} * \left(\frac{D_{m-m}}{2}\right)^2\right)\frac{q^2}{3}\right). \quad \text{(S3)}$$

On the other side, scattering intensity for a mixture at small q can be expressed by Guinier approximation in terms of apparent radius of gyration Rg_{mix}:

$$I(q) \equiv I(0)\left(1 - \frac{{Rg_{mix}}^2}{3} q^2\right). \quad \text{(S4)}$$

Thus, relation of gyration radius of the mixture, gyration radius of the monomer $Rg_1 \equiv Rg_{mono}$ and the inter-monomer distance between the mass centers within a dimer can expressed as:

$$Rg_{mix}^2 = Rg_{mono}^2 + \frac{2\alpha}{1+\alpha}\left(\frac{D_{m-m}}{2}\right)^2. \quad \text{(S5)}$$

Hence, transforming the equation (6), one can get an expression for D_{m-m}:

$$D_{m-m} = 2\sqrt{\frac{1+\alpha}{2\alpha}(Rg_{mix}^2 - Rg_{mono}^2)}. \quad \text{(S6)}$$

The Porod volume V of the particle can be calculated using the Porod invariant Q (see ref. 15 in the main text):

$$V_P = \frac{2\pi^2 I(0)}{Q} = \frac{2\pi^2 I(0)}{\int_0^{+\infty} I(q) q^2 \, dq}. \quad \text{(S7)}$$

For a mixture of various types of particles, particularly, in the case of a mixture of monomers and dimers, apparent Porod volume is equal to:

$$V_{P,mix} = \sum_j \alpha_j V_j = (1 + \alpha)\, V_{P,mono}. \quad \text{(S8)}$$

Volume fraction of the dimers α can be expressed from (8) as:

$$\alpha = V_{P,mix}/V_{P,mono} - 1. \quad \text{(S9)}$$
